# Design of the STRIVE-IPF Trial- Study of Therapeutic Plasma Exchange, Rituximab, and Intravenous Immunoglobulin for Acute Exacerbations of Idiopathic Pulmonary Fibrosis

**DOI:** 10.21203/rs.3.rs-3962419/v1

**Published:** 2024-02-28

**Authors:** Tejaswini Kulkarni, Gerard J. Criner, Daniel J. Kass, Ivan O. Rosas, Mary Beth Scholand, Daniel F. Dilling, Ross Summer, Steven R Duncan

**Affiliations:** 1 Department of Medicine, Division of Pulmonary, Allergy, and Critical Care Medicine, University of Alabama at Birmingham, Birmingham, Alabama, United States of America; 2 Department of Thoracic Medicine and Surgery, Lewis Katz School of Medicine at Temple University, Philadelphia, Pennsylvania, USA; 3 Division of Pulmonary, Allergy, and Critical Care Medicine, University of Pittsburgh, Pittsburgh, Pennsylvania, USA; 4 Section of Pulmonary, Critical Care and Sleep Medicine, Department of Medicine, Baylor College of Medicine, Houston, Texas, USA; 5 Pulmonary Division, Department of Internal Medicine, University of Utah, Salt Lake City, Utah, USA.; 6 Division of Pulmonary and Critical Care Medicine, Loyola University Chicago, Stritch School of Medicine, Maywood, IL; 7 Section of Pulmonary and Critical Care, Thomas Jefferson University, Philadelphia, PA

**Keywords:** Idiopathic Pulmonary Fibrosis, Acute exacerbation of Idiopathic Pulmonary Fibrosis, autoantibody reduction, clinical trial, protocol

## Abstract

**Background:**

Acute exacerbations of idiopathic pulmonary fibrosis (AE-IPF) affect a significant proportion of patients with IPF. There are limited data to inform therapeutic strategies for AEIPF, despite its high mortality. We discuss the rationale and design of STRIVE-IPF, a randomized, multi-center, open-label Phase IIb clinical trial to determine the efficacy of combined therapeutic plasma exchange (TPE), rituximab, and intravenous immunoglobulin (IVIG), in comparison to treatment as usual (TAU), among patients with acute IPF exacerbations.

**Methods:**

The STRIVE-IPF trial will randomize 51 patients among five sites in the United States. The inclusion criteria have been designed to select a study population with AE-IPF, as defined by American Thoracic Society criteria, while excluding patients with an alternative cause for a respiratory decompensation. The primary endpoint of this trial is six-month survival. Secondary endpoints include supplement oxygen requirement and six-minute walk distance which will be assessed immediately prior to treatment and after completion of therapy on day 19, as well as at periodic subsequent visits.

**Discussion:**

The experimental AE-IPF therapy proposed in this clinical trial was adapted from treatment regimens used in other antibody-mediated diseases. The regimen is initiated with TPE, which is expected to rapidly reduce circulating autoantibodies, followed by rituximab to reduce B-cells and finally IVIG, which likely has multiple effects, including affecting feedback inhibition of residual B-cells by Fc receptor occupancy. We have reported potential benefits of this experimental therapy for AE-IPF in previous anecdotal reports. This clinical trial has the potential to profoundly affect current paradigms and treatment approaches to patients with AE-IPF.

Trial Registration ClinicalTrials.gov identifier: NCT03286556

## Introduction:

Idiopathic pulmonary fibrosis (IPF) is a progressive fibroproliferative lung disease of older individuals (1). Patients with IPF have a median survival of only 3–5 years if untreated (2). Two antifibrotic therapies, nintedanib and pirfenidone, have been shown to slow loss of forced vital capacity (FVC) (3, 4). Nonetheless, a significant proportion of IPF patients, variously estimated as 5%−20% each year, develop acute exacerbations of their lung disease (AE-IPF) that are not attributable to other causes (5, 6). Because the etiology of AE-IPF is enigmatic, it has been impossible to rationally select agents that specifically target the underlying pathological mechanism(s). Further, there is significant heterogeneity in treatment practices for AE-IPF (7). No medical intervention yet tried has been shown to have efficacy for AE-IPF (5, 6, 8–10). The in-hospital mortality rate of patients with AE-IPF remains at nearly 50% and among those who survive their hospitalization, median survival following an acute IPF exacerbation is approximately 3 to 4 months (11, 12).

Recent studies have revealed several parallels between conventional autoimmune syndromes and progressive IPF (13–19). The role of immune processes in IPF has generally been discounted, primarily because this lung disorder does not respond to glucocorticoids. However, many other antibody-mediated lung diseases are similarly resistant or minimally responsive to steroids and nonspecific agents. Among these examples, granulomatosis with polyangiitis, Goodpasture’s syndrome, acute interstitial lung diseases (ILD) associated with polymyositis, rheumatoid arthritis (RA), or other classical autoimmune diseases, and lung transplant rejection due to alloantibodies, also have very high rates of progression and mortality when treated primarily (or solely) with steroids (20–25). In contrast, targeted treatments that reduce autoantibodies and/or prevent their generation are frequently beneficial for those syndromes.

The experimental AE-IPF therapy proposed in this clinical trial (Table 1) was adapted from treatment regimens used in other antibody-mediated lung diseases (20–26). We have previously reported that this regimen is well-tolerated by AE-IPF patients and seemed to be associated with improved gas exchange and prolonged survival in the majority of treated patients (27). Further, we also reported that AE-IPF patients who received this therapy and survived for one or more years had plasma anti-HEp-2 autoantibody titers that were approximately three-fold greater than those who succumbed.

The regimen is initiated with therapeutic plasma exchange (TPE), which rapidly reduces circulating autoantibodies (28, 29). Any single plasmapheresis only removes a minor proportion of total body autoantibodies, since concentrations of these immunoglobulins are greatest in extravascular tissues where they are inaccessible to extracorporeal filtration. Our current regimen consists of a total of nine TPE treatment given over two weeks. When this treatment was employed with fewer TPE over shorter periods, and although there was usually rapid initial benefit, fulminant relapses within 2–8 weeks were frequent (30). Hence, the multiple TPE sessions used now in this regimen result in greater and more beneficial depletion of pathogenic autoantibodies by allowing periods of re-equilibration of tissue immunoglobulins down a concentration gradient into the [accessible] plasma compartment (29). Rituximab reduces B-cells, the sources for subsequent autoantibody production. However, clinical benefits of rituximab accrue slowly over weeks to months (31), which prevents its isolated use, without prior TPE, in rapidly progressive AE-IPF patients. Finally, the regimen is completed with intravenous immunoglobulin (IVIG) which has many effects on autoantibody production, notably including feedback inhibition of residual B-cells by Fc receptor occupancy. This regimen appears to be an improvement compared to our results in AE-IPF patients who were treated with a shorter/simpler regimen (i.e., five TPE and rituximab, without IVIG) (30).

This manuscript describes the design of STRIVE-IPF, a clinical trial to test the use of this autoantibody reduction protocol, in acute IPF exacerbations (NCT03286556).

## Methods and analysis:

### Trial design

STRIVE-IPF is a randomized, multi-center, open-label Phase IIb clinical trial to determine the efficacy of combined TPE, rituximab, and IVIG, in comparison to treatment as usual (TAU), among patients with acute IPF exacerbations. A total of 51 subjects will be enrolled among five (5) sites in the Unites States. Subjects will be recruited from the inpatient populations of the collaborating institutions. The primary care physician/clinical care team, who may be study investigators or colleagues of the study investigators, will first identify potential research subjects and obtain their approval for discussion of the research project with the study investigators.

Following procurement of informed consent and completion of baseline/screening assessments, study subjects will be randomized 2:1 into one of two treatment arms: the combined TPE, rituximab, and IVIG (experimental therapy) vs. treatment as usual (TAU). TAU consists of empiric antibiotics and prednisone, as detailed below. All subjects, including those randomized to the experimental therapy arm will receive TAU. Subject eligibility will be confirmed prior to randomization by an adjudication process among the various participating investigators.

### Patient Population and Eligibility criteria

The inclusion criteria are intended to identify and study the patient population with AE-IPF, as defined by American Thoracic Society (ATS) criteria (6), while excluding patients with an alternative cause for a respiratory decompensation or with increased risk for the associated interventions. The eligibility criteria are listed in Table 2.

### Study Endpoints

The primary endpoint of this trial is six-month survival. All-cause mortality that occurs within 180 days of randomization will be tabulated. Lung transplantations that occur prior to 180 days will be treated as censored events, whereas institution of extracorporeal membrane oxygenation (ECMO) will be counted as an uncensored event (equivalent to death). Key clinical secondary endpoints include quantification of supplemental oxygen requirements at rest, and six-minute walk distance (6MWD). These secondary endpoints will be assessed immediately prior to treatment and after completion of therapy on day 19, as well as on days 60, 90, 180, 270, and 365. Subjects discharged from the hospital after completion of the protocol will be instructed to return to our clinics for these scheduled assessments. Adverse events and relapses that occur anytime during the subject’s participation in this study will be recorded and compared.

Supplemental oxygen measures, at the times specified above, will be tabulated as the flow rate (or concentration) necessary to maintain resting arterial oxygen saturations (per oximeter) at ≥93% (this gives some safety margin), as we used in the pilot trial (30). Many patients with AE-IPF cannot tolerate a tight-fitting face mask, nor breathe through a mouthpiece. Thus, we cannot systematically measure fraction of inspired oxygen (F_i_O_2_), which precludes rigorous analyses of P_a_O_2_:F_i_O_2_. Supplemental O_2_ requirements over the various intervals will be dichotomously categorized into one of two groups for comparison: 1) Improved (reduced by ≥50%) or 2) Unimproved (reduced <50%, unchanged or worsened). 6MWDs will be performed per ATS/ERS guidelines, by trained nursing staff in hospital or clinic hallways, our standard of practice (SOP). These 6MWD tests will also be assessed at the intervals listed above, and similarly dichotomized into either improved (by ≥30 meters) or unimproved (improved <30 meters, unchanged, or worsened). The cutoff value of 30 meters roughly corresponds to 6MWD minimal clinically important differences. The number of AE-IPF relapses (as described below) that occur in subjects in either study arm will be compared, adjusted for duration of exposure (i.e., deaths have potentially less time in which to have a relapse and thus this will be used in the analyses). Adverse events are a key secondary endpoint in this unblinded study and will be recorded as discussed in detail below. The key study endpoints are listed in table 3.

### Randomization

A randomization list, (2:1 experimental arm to controls) will be generated in advance by the Data Coordinating Center (DCC) and programmed into the web-based data entry and management system. Subjects will be stratified by disease severity as either A.) Mild (in order to maintain S_a_O_2_ ≥93%, supplemental O_2_ requirements are less than or equal to either 5L/min by nasal cannula or 50% O_2_ by face mask) or B.) Severe (supplemental O_2_ requirements > either 5L/min or >50% O_2_ per face mask). Subjects will also be stratified based on their use or nonuse of any/both antifibrotic medications (nintedanib and/or pirfenidone), maintaining a 2 to 1 randomization within strata. Due to the small sample size, stratification by site will not be attempted.

### Experimental Therapy Arm

#### Corticosteroids:

Subjects will be treated with 60 mg prednisone p.o. on day 1, followed by 20 mg prednisone/day p.o. thru day 19, except on days 6 and 15. Methylprednisolone (solumedrol) IV at equivalent doses may be used in lieu of prednisone. On days 6 and 15 subjects will receive 100 mg Solumedrol IV, as a premedication for rituximab. Steroid dosing after day 19 will be at the discretion of the attending physician. There is no evidence that any steroid regimen has efficacy for AE-IPF. Nonetheless, these agents are standard operating procedure (SOP) for AE-IPF patients with widely varying dosing regimens (5). The experimental trial subjects will also have a dialysis/pheresis catheter placed in a central vein for their TPE.

#### Therapeutic Plasma Exchange:

TPE consist of 1x estimated plasma volume exchanges for 3 successive days (days 1,2, and 3), followed by two more daily treatments on days 5 and 6, and then four more days (9,11,13, and 15). Initial daily TPE is intended to promptly decrease autoantibody titers in these often rapidly progressive subjects. Subsequent interrupted scheduling will allow equilibration of tissue-bound autoantibodies into the circulation, thereby increasing TPE efficacy. The 48-hour interruption (days 7–8) is to enable the first dose of slow-onset rituximab to be administered early, rather than later (after all TPE). Spacing of 48 hrs between rituximab and the next TPE reduces removal of the anti-B-cell agent through TPE.

Plasma fluid volume will be replaced during TPE with 5% albumin, to maintain a net fluid balance of 95–100%. In the event a subject’s fibrinogen is <120 mg/dL at the initiation of a TPE, as may occur in later treatments, either (1) cryoprecipitated antihaemophilic factor (AHF) (two 5-pooled units) will be given prior to the procedure to raise the fibrinogen level or (2) the replacement fluid will be 50% plasma and 50% 5% albumin, in order to maintain post-procedural fibrinogen >50 mg/dL and thus, minimize bleeding risks. The use of cryoprecipitated AHF prior to the procedure or plasma as the replacement fluid during the procedure will be at the discretion of the attending apheresis physician. Calcium supplementation, either by PO or by IV, may be given to reduce citrate toxicity related to TPE. Dialysis catheters are removed after the last TPE, as long as the fibrinogen level is >100 mg/dL.

#### Rituximab:

One gram IV will be administered on day 6 (after completion of that day’s TPE) and again on day 15, after the last TPE. Subjects will be premedicated with acetaminophen, diphenhydramine, and methylprednisolone 100 mg IV to obviate reactions.

#### Intravenous immunoglobulin:

IVIG doses of 0.5 gm/kg will be administered on days 16–19, after the last TPE. Subjects who have mild reactions will be premedicated, as with rituximab, prior to subsequent doses. This is also SOP for lung transplant recipients treated with IVIG for antibody-mediated allograft rejection in our hospitals.

#### Empiric Antibiotics:

All subjects, at all sites, in both treatment arms, will receive antibiotics for 8 days, a SOP for empiric therapy of patients with exacerbations of chronic lung diseases, based on recognition that bronchoalveolar lavage (BAL) and transtracheal aspiration (TTA) have poor diagnostic accuracy. Empiric antibiotic therapy for AE-IPF is widely accepted. The 8-day duration is based on data in other seriously ill patients (32). Our SOP regimen is: azithromycin (until *Legionella* urine DFA is negative) plus piperacillin/tazobactam + vancomycin. Substitutions for allergies are ciprofloxacin and linezolid, respectively. Antibiotics will be administered as specified by package inserts. Subjects intubated after enrollment will be managed using standard ARDS Network guidelines.

The duration of this antibiotic regimen can be shortened, at the discretion of the attending physician, among patients who have been treated with an identical (or clinically comparable) regimen, at the time of or immediately prior to enrollment in STRIVE-IPF (e.g., patients who were getting antibiotics at an outside hospital prior to transfer to the study site). Given difficulties in diagnosing infections in these often critically ill patients, the AE-IPF subjects in this trial should receive appropriate antibiotics, but consideration for adjustment is necessary in cases in which the total duration of antibiotic therapy would be excessive and potentially deleterious.

#### Treatment as usual:

All subjects, at all sites, in both treatment arms, will also receive: 60 mg prednisone PO on day 1, followed by 20 mg prednisone/day PO thru day 19, except on days 6 and 15. Methylprednisolone IV at equivalent doses may be used in lieu of prednisone. On days 6 and 15 subjects will receive 100 mg Solumedrol IV. Steroid dosing after day 19 will be at the discretion of the attending physician.

### Study duration and follow-up

The treatment protocol will last for 19 days. Enrolled patients may be discharged from the hospital prior to the completion of the 19-day therapy, if medically indicated per their attending physicians, and if they can still receive their study treatments and maintain protocol compliance as outpatients. Conversely, enrolled subjects still hospitalized on day 20 will be followed for the duration of their hospital stay. Patients removed from the study for unacceptable adverse events will be followed until resolution or stabilization of the adverse event. Subjects discharged from the hospital will be encouraged to return as outpatients for observations and specimen acquisitions on days 60, 90, 180, 270, and 365. Telephone interview to detect potential late complications and outcomes will be conducted by study coordinators at other monthly intervals. The detailed study procedures and timeline are listed in Table 4.

### Relapse among subjects

A relapse is defined as a recrudescence of AE-IPF symptoms and physical findings, i.e., new or increased shortness of breath or hypoxemia during the last 30 days, without other explicable etiology, after a favorable clinical response to initial therapy (as distinct from progressive, treatment refractory episodes). The definition of a “favorable clinical response” is based on our clinical secondary endpoints, and consists of either a reduction in requirement for supplemental oxygen by 50% or an improvement of 6MWD by 30 meters lasting for at least 5 days after completion of their initial treatment The presence of a relapse will be confirmed by adjudication among the PIs, following the same communication and survey procedures used to adjudicate and confirm the initial diagnoses of AE-IPF.

The treatment of an AE-IPF relapse in a participant is based on their initial randomization and treatment. All subjects will again be treated with 60 mg prednisone PO (or methylprednisolone equivalent) on day 1, followed by 20 mg/day for the duration of their therapy, and the specified empiric antibiotic regimen. In addition, experimental arm subjects who relapse will receive TPE × 5, administered every other day, followed by four successive doses of IVIG (0.5 g/kg/day). TAU subjects will again be treated with the steroids and antibiotics. Second relapses in any treatment arm, if these occur within the 6-month trial participation, will be treated with standard therapy (i.e., TAU).

### Adverse Events

Patients will be monitored carefully for occurrence of adverse events (AEs), laboratory test abnormalities, and changes in vital signs. Adverse events will be evaluated according to criteria outlined in the NCI Common Terminology Criteria for Adverse Events (CTCAE, version 4.0) (33). The NCI Common Toxicity Criteria Scale will be used to define grades (severity) of adverse events and toxicities. An adverse event is defined as any untoward medical occurrence in a subject, regardless of its relationship to these treatments. Toxicity is an adverse event with a direct relationship to the study drug. All toxicities are adverse events, but not all adverse events are toxicities. Computer data entry (and case report forms- [CRF]) will require responses to all adverse events, with a particular focus on infections (including location and organism), catheter-related problems (e.g., bleeding, clot), apheresis-related complications (e.g., hypotension, citrate toxicity, etc), metabolic or hemodynamic perturbations (e.g., hyperglycemia, hypotension, etc.) and other, less frequent complications (e.g., neurologic symptoms), as well as having provision for free text entries. Recording all AEs in pre-specified checklists (and free text entries), will guard against unintended bias in this unblinded trial.

### Statistical Analysis

Baseline demographic and clinical data of participants will be summarized using means (with standard deviations) and medians (with inter-quartile ranges) for continuous variables, and counts and percentages for discrete variables, stratified by treatment group. Differences among groups (to examine the randomization effectiveness), will be assessed by t-tests, or by Fisher’s exact test for discrete data. Deaths will be tabulated based on the actual day they occur, relative to the date of randomization. Analysis will be based on intention-to-treat and deaths at any time will be counted against the treatment arm assigned. Survival analyses will use an exponential survival model and be summarized using Kaplan-Meier plots via product-limit estimation and log-rank tests. Lung transplantations, or institution of extracorporeal membrane oxygenation (ECMO) will be used as events in sensitivity analyses to ensure the consistency of the outcome results, since these are likely informative censorings.

For the secondary analyses, we will use repeated measures mixed effects models to utilize the maximum data from each participant, in addition to the dichotomized (improved vs. otherwise) assessments for oxygen requirements and walk distance (to follow). The proportion of subjects with significant O2 and 6MWD changes in each arm will be compared by Fisher’s exact test. For sensitivity analyses, we will use the worst O2 from a completing patient for any deaths within each treatment group. Similarly for the 6-minute walk we will impute the worst time within the treatment arm surviving to complete the walk. For the AEs and relapses, we will use Poisson regression with an offset for the time under study.

### Data and Safety Monitoring Board

An independent data monitoring committee will perform regular review of the data, particularly those relating to serious AEs, or AEs leading to discontinuation of study drug and laboratory parameters. The DSMB will meet approximately 2 times per year to review safety and overall study progress until the end of the study.

## Discussion:

AE-IPF is characterized by clinically significant respiratory deterioration, defined as either (and usually both) increased dyspnea and/or oxygen requirement, with onset during the preceding month, and with radiographic evidence of infiltrates, superimposed on classic fibrotic abnormalities. The development of AE-IPF portends a poor prognosis (6). There is a lack of consensus on current therapies for AE-IPF and recommendations, are based on anecdotal consensus statements and small, uncontrolled, or retrospective studies (9). Major hurdles to developing effective therapies for AE-IPF include its apparently complex pathobiology and many uncertainties understanding the triggers for an AE.

### Challenges to designing an AE-IPF trial

In contrast to multiple past and ongoing trials of therapies for IPF outpatients, only two randomized clinical trials (RCTs) have specifically enrolled AE-IPF patients (34, 35). Thrombomodulin alfa was used in this indication based on its anticoagulant and anti-inflammatory effects. However, the drug did not result in an improvement of the 90-day survival (72.5% in the thrombomodulin alfa group vs 89.2% in the placebo group) (34). The experimental treatment in the other RCT, consisted of intravenous pulses of cyclophosphamide added to high-dose glucocorticoids, and was associated with a trend towards increased all-cause mortality at 3 months, compared to high-dose glucocorticoids and placebo (45% vs 31%, difference 14·5% [95% CI −3·1 to 31·6]; p=0·10) (35).

Of note, subjects in both trials received high-dose glucocorticoids due to concerns about recruitment and ethical challenges for a trial with a true placebo arm. More recent data have shown evidence that patients treated with high dose glucocorticoids may have worse outcomes (36). After consideration of these concerns and evaluation of the available data, we elected to treat all patients in STRIVE-IPF with prednisone 20mg (or equivalent).

An important consideration for the STRIVE-IPF study is the open-label design. Although blinding is in most cases the optimal design to eliminate bias, we believed that requiring subjects in the TAU to undergo sham central venous catheter placements that are associated with discomfort, potential morbidity, and finite (if small) mortality was unethical. Furthermore, there is no facile way to blind for the TPE treatment *per se* and no sham machines are available. Nonetheless, inadvertent bias should be minimal, given the unequivocal primary endpoint (i.e., 6-month survival).

### Rationale for study endpoints

Patient survival, the primary endpoint of this trial, is unarguably important, objective, and accurately ascertained from observations, patient or family phone interviews, and internet obituary searches. Secondary endpoints reflect parameters of clinical improvement with treatment-related effects on supplemental O2 requirements and 6MWD. Establishing safety of the experimental therapy is of utmost importance. FVC is an established efficacy parameter of disease progression in IPF and has served as the basis for worldwide regulatory approvals for nintedanib and pirfenidone for treatment of IPF. While FVC reflects a clinically meaningful outcome in the outpatient setting, patients with AE-IPF often have difficulty performing the test while hospitalized due to severe dyspnea and requirements for high concentrations of supplemental oxygen. Furthermore, FVC during an acute inpatient stay could be independently reduced through other mechanisms (fluid shifts, atelectasis, muscle weakness) and FVC measurement is not part of a routine assessment of AE-IPF evaluation or therapeutic response.

### Recruitment challenges in an AE-IPF trial

The annual incidence of AE-IPF is estimated variously at 5–20% and, as such, is an infrequent complication of a rare disease (6). Compared to the recruitment rate in traditional RCTs for IPF, the accrual rate for AE-IPF can be much lower due to the much lesser incidences and confounding clinical conditions of the patients. Another significant recruitment challenge is our desire to excluded patients with positive (abnormal) standard autoimmune tests: antinuclear antibody (ANA), rheumatoid factor (RF), anti-SSA, and anti-CCP in order to eliminate all later considerations that our study population includes patients with autoimmune syndromes, rather than IPF. The prevalence of one or more positive values of these autoantibody tests (often at barely abnormal titers) may be as great as 45% patients with IPF and no other features of autoimmunity (37, 38). We anticipated a priori that many IPF patients would be excluded due to “false positive” autoantibody tests but believe our focus on treatment of a population with unequicoval diagnoses will lend rigor to our trial results.

## Conclusion:

AE-IPF is a highly lethal syndrome for which no therapy has proven benefit. The results of this trial could provide compelling evidence that autoantibody reduction therapy benefits AE-IPF patients. This trial has the potential to be paradigm-shifting, alter current approaches to AE-IPF treatment, and could ultimately reduce mortality in patients with this dismal presentation.

## Figures and Tables

**Table 1:** Outline of Experimental Arm Interventions

Days on Intervention	1	2	3	4	5	6	7	8	9	10	11	12	13	14	15	16	17	18	19
**Prednisone or equivalent (p.o. dose per day)**	**60 mg**	**20 mg each day**				**none**	**20 mg each day**								**none**	**20 mg each day**			
**Therapeutic Plasma Exchange (TPE)**	**TPE, each of these days**				**TPE, each of these days**				**TPE**		**TPE**		**TPE**		**TPE**				
**Rituximab**						**1 gm IV**									**1gm IV**				
**Acetaminophen** (prior to Rituximab)						**650 mg Oral**									**650 mg Oral**				
**Solumedrol** (prior to Rituximab)						**100 mg IV**									**100 mg IV**				
**Benadryl -** diphenhydramine (prior to Rituximab)						**50 mg Oral**									**50 mg Oral**				
**Antibiotics**	**x**	**x**	**x**	**x**	**x**	**x**	**x**	**x**											
**IVIG**																**0.5 gm/kg/day**			

The experimental interventions (days 1–19) are outlined here.

Maintenance steroid doses after Day 19 are at discretion of attending physician(s). Adverse events are prospectively recorded at these time points too, and at any time they may arise.

**Table 2:** Study Eligibility Criteria

Inclusion Criteria	Age between 40–85 years old. A diagnosis of IPF that fulfills ATS/ERS Consensus Criteria.1 Hospitalized patient with a diagnosis of AE-IPF, as ascertained by the responsible Primary Investigator, with findings that include worsening or new development of dyspnea or hypoxemia within the last 30 days. Ground-glass abnormality and/or consolidation superimposed on a reticular or honeycomb UIP pattern on locally read chest CT scan. Ability and willingness to give informed consent and adhere to study requirements.
Exclusion Criteria	Diagnoses of current infection per clinical or microbial assessments. Diagnoses of an additional or alternative etiology for respiratory dysfunction based upon clinical assessment, including congestive heart failure, sepsis, thromboembolism, etc, History or serological evidence of hepatitis B, or current hepatitis C infection. Coagulopathy, defined as an INR >1.6, PTT > 2x control, fibrinogen <100 mg/dL, or platelet count <50K unless these abnormalities can be reversed safely. Hyperosmolar state or diabetic ketoacidosis to suggest uncontrolled diabetes, or uncontrolled hypertension (systolic BP >160 mm Hg and diastolic BP >100 mm Hg) that would contraindicate use of corticosteroids. Hemodynamic instability, defined as an inotrope or vasopressor requirement. History of reaction to blood products or murine-derived products. Active malignancy or undergoing treatment of malignancy, excluding basal or squamous cell skin cancer and low-risk prostate cancer, the latter defined as stage T1 or T2a, with prostate specific antigen (PSA) less than 10 ng/dl. The experimental treatments are not known to promote cancer progression, and these criteria are within current guidelines. Unwillingness to accept blood product transfusion. Diagnosis of major comorbidities, or other considerations, that the responsible physicianinvestigators believe should disqualify subjects or are expected to interfere with study participation. Treatment for >14 days within the preceding month with >20 mg. prednisone (or equivalent) or any treatment during the last month with a cellular immuno-suppressant (e.g., cyclophosphamide, methotrexate, calcineurin inhibitors, mycophenolate, azathioprine, etc.). An exception will be made if the patient has a bronchoalveolar lavage (BAL) negative for pathogens. Presence of a condition or ongoing treatment with a medication that precludes TPE. Concurrent participation in other experimental trials. Fertile females who do not agree to contraception or abstinence or have a positive pregnancy test (urine or blood). IPF is a disease of older adults, and male predominant, so this will not be a frequent consideration. Presence of positive (abnormal) classical autoimmune tests: ANA, RF, Anti-SSA, and AntiCCP. This criterion will eliminate patients with confounding classical autoimmune syndromes. Many IPF patients will have already had these tests, which are standard of practice at many IPF centers, and these prior results will suffice if the tests were performed within the last year. Otherwise, these tests need to be performed prior to enrollment and they can usually be procured in 1–2 days. ANCA will continue to be tested during screening, but negative results are no longer a requirement for randomization. Based on experience, we anticipate ~10% of patients who fulfill all other IPF criteria will nonetheless be positive for one of these classical autoantibody tests. IgA deficiency (IgA level <7 mg/dL)- to preclude IVIG reactions. Listed with the United Network for Organ Sharing for lung transplant at the time of enrollment. Patients who are intubated at time of STRIVE enrollment. Prior use of rituximab within the year preceding enrollment. Clinical or Morphologic Domain criteria of Idiopathic Pulmonary Fibrosis with Autoimmune features.

**Table 3.** Key Study End-points

Primary Endpoint	Secondary Endpoints	Exploratory Endpoints
Survival at 6 months	Supplemental O2 requirements	Anti-HEp-2 IFA Titers and Patterns
	Six-minute walk distance	Circulating Immunoglobulin Concentrations
	AE-IPF Relapses	B Lymphocyte Stimulating Factor
	Adverse events	Quality of Life (QoL) Measures
		Autoantibody repertoires

AE-IPF: Acute exacerbations of Idiopathic Pulmonary Fibrosis, IFA: Indirect Immunofluorescence Assay

**Table 4:** Study Procedures

Days on Intervention	Screening	1	2	3	5	6	9	11	13	15	19	60	90	180	270	365
**Informed Consent**	**X**															
**Demographics**	**X**															
**Medical History**	**X**											**X**	**X**	**X**	**X**	**X**
**Eligibility Review**	**X**															
**Symptom Assessment**	**X**											**X**	**X**	**X**	**X**	**X**
**Complete Physical Exam**	**X**											**X**	**X**	**X**	**X**	**X**
**Vital signs**	**X**											**X**	**X**	**X**	**X**	**X**
**Routine Blood tests**	**X**	**X**														
**Protocol Specific Tests** #	**X**															
**Pregnancy Test (if applicable)**	**X**															
**Experimental Arm Only**																
Experimental Blood Specimens		Before therapy										**X**	**X**	**X**	**X**	**X**
Routine Blood tests	**X**	**X**	**X**	**X**	**X**	**X**	**X**	**X**	**X**	**X**						
O_2_ requirement assessment		Before therapy									After therapy	**X**	**X**	**X**	**X**	**X**
TPE Collection		500 ml + 50 ml		50 ml			50 ml			50 ml						
6- minute walk distance		Before therapy									After therapy	**X**	**X**	**X**	**X**	**X**
**Treatment as Usual Arm Only**																
Experimental Blood Specimens		**X**										**X**	**X**	**X**	**X**	**X**
O_2_ requirement assessment		**X**									X*	**X**	**X**	**X**	**X**	**X**
6- minute walk distance		**X**									X*	**X**	**X**	**X**	**X**	**X**
**Adverse Event Monitoring**	**X**	**X**	**X**	**X**	**X**	**X**	**X**	**X**	**X**	**X**	**X**	**X**	**X**	**X**	**X**	**X**

# Laboratory evaluations specific for Inclusion/Exclusion Criteria to include: hepatitis B surface antigen (HBsAg), hepatitis B core antibody (HBcAb) and hepatitis C virus antibody (HCV Ab). tests, IgA, ANA, RF, anti-SSA, anti-CCP.

* At discharge, discretion of the treating physician. Experimental immunologic assays (blood samples to be processed and stored frozen), for later batch shipment (while frozen) to the Coordinating Center. Telephone contacts (end of months 1, 4, 5, 7, 8, 10, 11).

## Data Availability

Not applicable to this protocol paper

## List of Abbreviations:

IPF: Idiopathic Pulmonary Fibrosis
AE-IPF: Acute Exacerbations of Idiopathic pulmonary fibrosis
ILD: Interstitial Lung Diseases
RA: Rheumatoid arthritis
TPE: Therapeutic Plasma Exchange
IVIG: Intravenous Immunoglobulin
TAU: Treatment as Usua
ATS: American Thoracic Society
6MWD: Six-Minute Walk Distance
FiO2: Fraction of Inspired Oxygen
ERS: European Respiratory Society
DCC: Data Coordinating Center
IV: Intravenous
SOP: Standard Operating Procedure
PO: per os
BAL: Bronchoalveolar Lavage
PI: Primary Investigator
CRF: Case Report Forms
AEs: Adverse Events
ECMO: Extracorporeal Membrane Oxygenation
DSMB: Data and Safety Monitoring Board
RCT: Randomized Clinical Trials
